# Ultrasound-Based Molecular Imaging of Tumors with PTPmu Biomarker-Targeted Nanobubble Contrast Agents

**DOI:** 10.3390/ijms22041983

**Published:** 2021-02-17

**Authors:** Mette L. Johansen, Reshani Perera, Eric Abenojar, Xinning Wang, Jason Vincent, Agata A. Exner, Susann M. Brady-Kalnay

**Affiliations:** 1 Department of Molecular Biology and Microbiology, School of Medicine, Case Western Reserve University, 10900 Euclid Ave, Cleveland, OH 44106-4960, USA; mette.johansen@case.edu (M.L.J.); jason.vincent@case.edu (J.V.); 2 Department of Radiology, Case Western Reserve University, Cleveland, OH 44106-4960, USA; reshani.perera@case.edu (R.P.); eric.abenojar@case.edu (E.A.); 3 Department of Biomedical Engineering, Case Western Reserve University, Cleveland, OH 44106-7207, USA; xinning.wang@case.edu

**Keywords:** nanobubbles, PTPmu, receptor protein tyrosine phosphatase, molecular imaging, dynamic contrast enhanced ultrasound, ultrasound contrast agents, time intensity curve

## Abstract

Ultrasound imaging is a widely used, readily accessible and safe imaging modality. Molecularly-targeted microbubble- and nanobubble-based contrast agents used in conjunction with ultrasound imaging expand the utility of this modality by specifically targeting and detecting biomarkers associated with different pathologies including cancer. In this study, nanobubbles directed to a cancer biomarker derived from the Receptor Protein Tyrosine Phosphatase mu, PTPmu, were evaluated alongside non-targeted nanobubbles using contrast enhanced ultrasound both in vitro and in vivo in mice. In vitro resonant mass and clinical ultrasound measurements showed gas-core, lipid-shelled nanobubbles conjugated to either a PTPmu-directed peptide or a Scrambled control peptide were equivalent. Mice with heterotopic human tumors expressing the PTPmu-biomarker were injected with PTPmu-targeted or control nanobubbles and dynamic contrast-enhanced ultrasound was performed. Tumor enhancement was more rapid and greater with PTPmu-targeted nanobubbles compared to the non-targeted control nanobubbles. Peak tumor enhancement by the PTPmu-targeted nanobubbles occurred within five minutes of contrast injection and was more than 35% higher than the Scrambled nanobubble signal for the subsequent two minutes. At later time points, the signal in tumors remained higher with PTPmu-targeted nanobubbles demonstrating that PTPmu-targeted nanobubbles recognize tumors using molecular ultrasound imaging and may be useful for diagnostic and therapeutic purposes.

## 1. Introduction

The use of ultrasound (US) imaging for diagnostic purposes has grown considerably in recent years due to its wide availability, its adaptability, its safety profile and high sensitivity [1,2]. Recent data show that the annual number of ultrasound imaging procedures was second only to diagnostic X-rays and far surpassed the number of computed tomography (CT) scans, which was the third most common diagnostic imaging modality [1]. Advantages of US relative to X-ray, CT, and positron emission tomography (PET) scans include the absence of patient exposure to any ionizing radiation and the real time conveyance of information to clinicians [1,3,4]. Compared to magnetic resonance imaging (MRI) and CT/PET scanners, US instruments are less expensive and more portable, qualities that contribute to cost-effective diagnostic applications with equivalent or improved accuracy in many applications [5].

Ultrasound contrast agents (UCAs) are used to enhance US imaging and can be adapted for therapeutic and theranostic uses [2,3,4,6,7,8]. Currently, commercially available UCAs are categorized as microbubbles (MBs) with sizes ranging from 1 to 10 µm and consist of phospholipid or albumin shells that enclose an echogenic perfluorocarbon gas [2,3,4,6,8,9]. MBs act as blood pool contrast agents since their size prevents their transit out of the blood stream [2,3,4,6,8,9]. Relative to contrast agents used in MRI and PET/CT which may be nephrotoxic, hepatotoxic, and/or radioactive, UCAs are considered very safe [1,3,6]. UCAs have been used worldwide in clinical applications for over 20 years, although the number and types of approved applications differ by country [4,6]. In the United States, FDA-approved MBs for echocardiography have been available since the 1990s and are used primarily to improve delineation of the left ventricular endocardial border, which is achieved by enhancing or “opacifying” the blood within it in a procedure termed “left ventricle opacification (LVO)” [1,4,6,10]. The ability to monitor blood flow in real-time also makes it possible for clinicians to use these UCAs in assessing myocardial perfusion following suspected cardiac events [1,4,6,10]. More recently, approved applications in the United States include the use of UCAs to examine liver lesions and for voiding urosonography [4,6], applications that underscore the advantages of being able to dynamically image fluid flow. In liver, contrast enhanced US (CEUS) can be used to distinguish between different types of malignant and benign lesions based on the patterns of phase enhancement (reviewed in [4,6]), and may provide a cost-effective alternative to the more standard use of MRI. MBs have also shown preclinical utility in imaging kidney lesions and in assessing perfusion in recently transplanted kidneys [4,6].

Recent advances in UCA technology include the development of molecular UCAs, which are agents with targeting moieties that recognize and bind to molecules upregulated on specific tissues or cells during pathologic states such as cancer or inflammation (reviewed in [2,7,8]). Some examples include UCAs targeted to prostate specific membrane antigen (PSMA) for use in imaging prostate cancer [11,12], and BR55, a MB-based agent targeted to vascular endothelial growth factor receptor 2 (VEGFR2), a hallmark of tumor-associated angiogenesis [13,14]. Along with increasing specificity, investigations are also underway to adapt UCAs for therapeutic use [7,15,16,17]. Several strategies have been employed to take advantage of the unique attributes of US and UCAs for applications such as drug delivery, transient opening of the blood brain barrier and ablation [7,15,16,17]. In addition to these refinements in MB design, an active area of UCA research involves the development of nanobubbles (NBs) to complement the use of MBs and/or expand the potential for therapeutic and theranostic applications. NBs are particles <1 µm (generally 100–500 nm) in diameter and have a structure similar to MBs, with a phospholipid or polymer shell with a hydrophobic gas core. In contrast to MBs, NBs are small enough to transit out of the blood stream [2,3,8,12,15] thus making more tissues accessible to NB-based theranostics. As with MBs, targeting molecules conjugated to the NB surface further extend the utility of NBs by making them specific for tumors or other target cells and tissues. The objective of this study was to utilize a highly stable NB formulation [11,18] with a new targeting strategy using a Receptor Protein Tyrosine Phosphatase mu (PTPmu/PTPµ) targeting peptide, SBK2. We evaluated the specificity of PTPµ-targeted NBs compared to non-targeting NBs with a non-specific scrambled peptide in an in vivo tumor model.

PTPµ is a member of the receptor protein tyrosine phosphatase family and is important for mediating cell-cell adhesion via homophilic interactions. In healthy cells, Mephrin/A5-protein/PTPmu (MAM) and immunoglobulin domains in the extracellular region of the molecule mediate binding events between adjacent PTPµ molecules on the cell surface [19,20,21]. As with other adhesion molecules, oligomeric PTPµ molecules are thought to cluster within the plane of the cell membrane of one cell to form tight adhesions with PTPµ clusters on an adjacent cell. In contrast to healthy cells, aberrant, increased protease activity is often observed in tumor cells [22,23,24]. In the tumor setting, proteolysis of adhesion molecules disrupts cell-cell and cell-matrix adhesions, alters signaling events needed for contact inhibition and increases migratory behavior. We have previously shown that PTPµ is susceptible to cleavage in the tumor microenvironment and proteolysis of PTPµ contributes to enhanced growth and migration of tumor cells [25,26,27]. Proteolytic targeting of PTPµ leads to the disruption of normal PTPµ-PTPµ interactions and results in the formation of an extracellular PTPµ fragment and thus a tumor specific biomarker. In previous work, we took advantage of the homophilic nature of PTPµ binding to identify PTPµ-derived peptides that can be used to detect this PTPµ tumor biomarker when coupled to fluorophores [25]. In vitro studies using Texas Red-labeled PTPµ peptides demonstrated the presence of the PTPµ biomarker in tumor sections but not in normal tissues [25]. More recently, we found that in brain tumor patients, the PTPµ biomarker was a prognostic indicator [28]. Ex vivo studies have further illustrated the remarkable ability of these peptides to label the biomarker on invasive tumor cells that have migrated far away from the main tumor [29,30]. In light of these studies, we have generated PTPµ-targeted NBs and characterized their ability to target and enhance the detectable US signal in tumors.

## 2. Results

**Figure 1:**
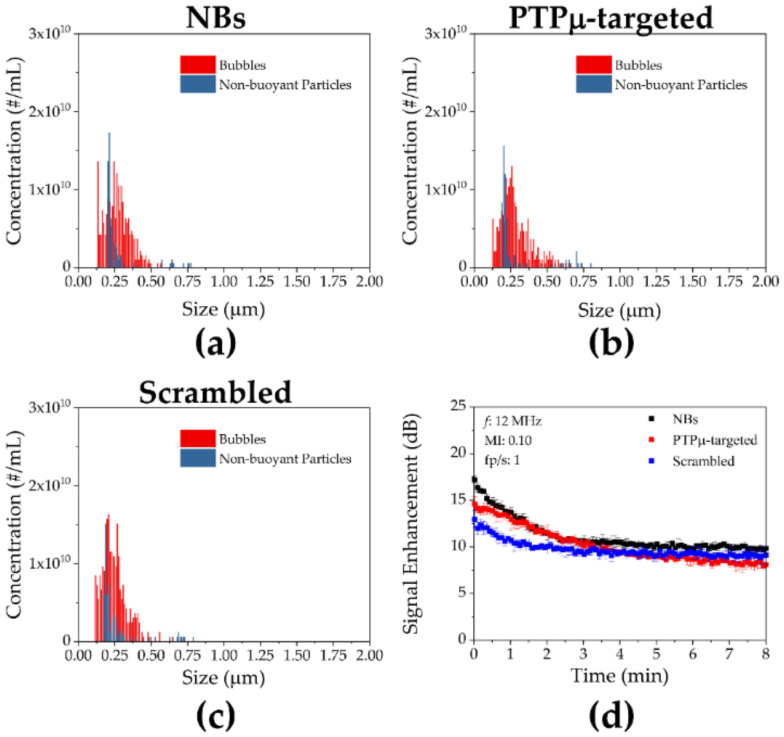
In vitro characterization of nanobubbles. The size and concentration distributions for (**a**) non-peptide modified nanobubbles (NBs), (**b**) Receptor Protein Tyrosine Phosphatase mu (PTPµ)-targeted NBs and (**c**) Scrambled NBs measured using Archimedes resonant mass measurement are shown. (**d**) In vitro ultrasound characterization of the three different NB types. No significant differences were seen between the formulations.

Nanobubbles incorporating the PTPµ-targeting peptide SBK2 or a scrambled version of the peptide (“Scrambled”), as non-targeting control NBs, were first compared in vitro. As shown in Figure 1, the PTPµ-targeted and Scrambled NBs demonstrated size and concentration distributions that were nearly identical to the size and buoyancy distribution of NBs made without any peptide-conjugated lipids (“Plain”). The mean size and standard deviations were 272 ± 89 nm, 285 ± 104 nm and 248 ± 85 nm for Plain, PTPµ-targeted and Scrambled NB, respectively, for the representative preparations shown in Figure 1. The corresponding concentrations for these NBs were 2.03 × 10^11^, 1.99 × 10^11^ and 2.38 × 10^11^ NB/mL. There were no significant differences in size or concentration for any of the NBs. All three NB types also showed comparable stability when subjected to contrast harmonic imaging in vitro (Figure 1d). As shown in Figure 1d, the in vitro US signals varied slightly initially, but by 3 min into the scans, all three NB types (Plain, PTPµ-targeted and Scrambled) showed US signals of approximately 10 dB which were maintained throughout the scan time.

To ascertain baseline in vivo characteristics of the targeted and untargeted NBs, non-tumor bearing nude mice (*N* = 3) were injected with 100 µL of one NB type (2 × 10^11^ NBs/mL) and US signals were collected simultaneously from the heart, liver and kidney for 30 min. After 30 min, residual NBs still present were destroyed by 3 high intensity ultrasound bursts, the animals were maintained under anesthesia for an additional 30 min to ensure clearance of all NBs and then 100 µL of the alternate NB type was injected and imaging of the heart, kidney and liver repeated for 30 min.

**Figure 2:**
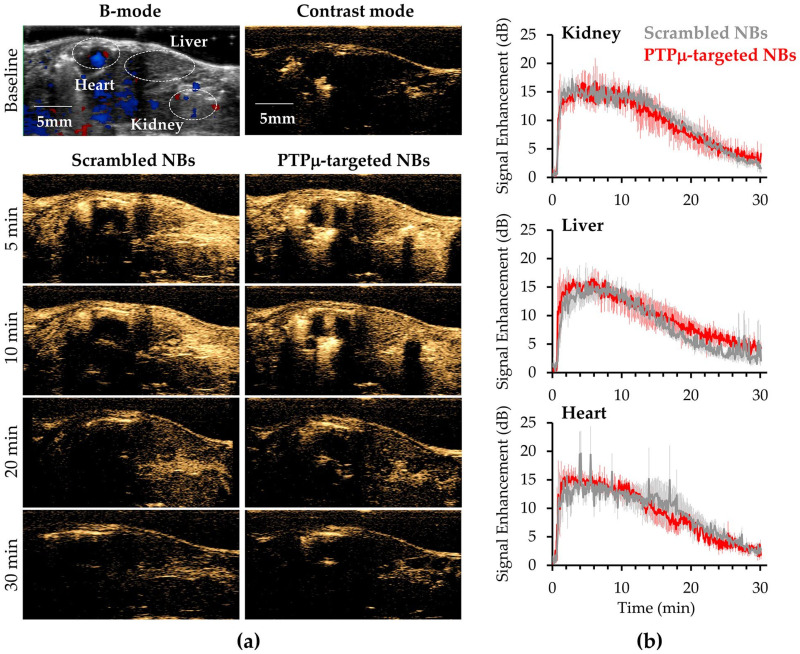
Ultrasound imaging of non-tumor bearing control mice treated with Scrambled and PTPµ-targeted NBs. (**a**) Representative images of non-tumor bearing control mice administered Scrambled-NBs and PTPµ-targeted NBs. The two NB types show similar enhancement of the heart, liver and kidneys at the times indicated. Top row shows a B-mode baseline image used for positioning (left) and a baseline contrast harmonic image (CHI) (right). Lower panels show heart, liver and kidney enhancement by the different NBs at the indicated times. (**b**) PTPµ-targeted and non-targeted NB (Scrambled-NB) show similar kinetics of signal enhancement and gradual clearance in kidney, liver and heart in non-tumor bearing mice. Images of the three organs were collected simultaneously over 30 min in mice (*N* = 3) receiving both types of NB. Mean signal intensity ± standard error are plotted for each organ type.

As shown in Figure 2, the two NB versions showed nearly identical signal enhancement and clearance in each of the three organs examined in the non-tumor bearing mice. Following injection, NB signals from either NB species were visible within 5 s in all three organs and peak contrast occurred by 5 min following the start of either NB injection. A gradual decrease in signal was observed in all three organs starting around 10 min and continued to decrease throughout the 30 min imaging window (Figure 2). At 30 min, some signal above baseline was still evident for both types of bubbles in all three organs, which demonstrates similar in vivo stability and clearance in non-tumor bearing mice. As shown in Figure 2a, both NB types rapidly filled all tissues including the kidney, liver and heart. Analysis of time-intensity curves revealed slightly different values of maximum peak intensity among the three organs but the differences were not significant (Figure 2b). The maximum peak intensities reached by the Scrambled and PTPµ-targeted NBs, respectively, were 19.3 ± 0.6 dB and 20.4 ± 2.1 dB in kidney, 17.3 ± 2.5 dB and 19.0 ± 0.7 dB in liver and 18.2 ± 1.6 dB and 18.4 ± 1.3 dB in heart.

**Figure 3:**
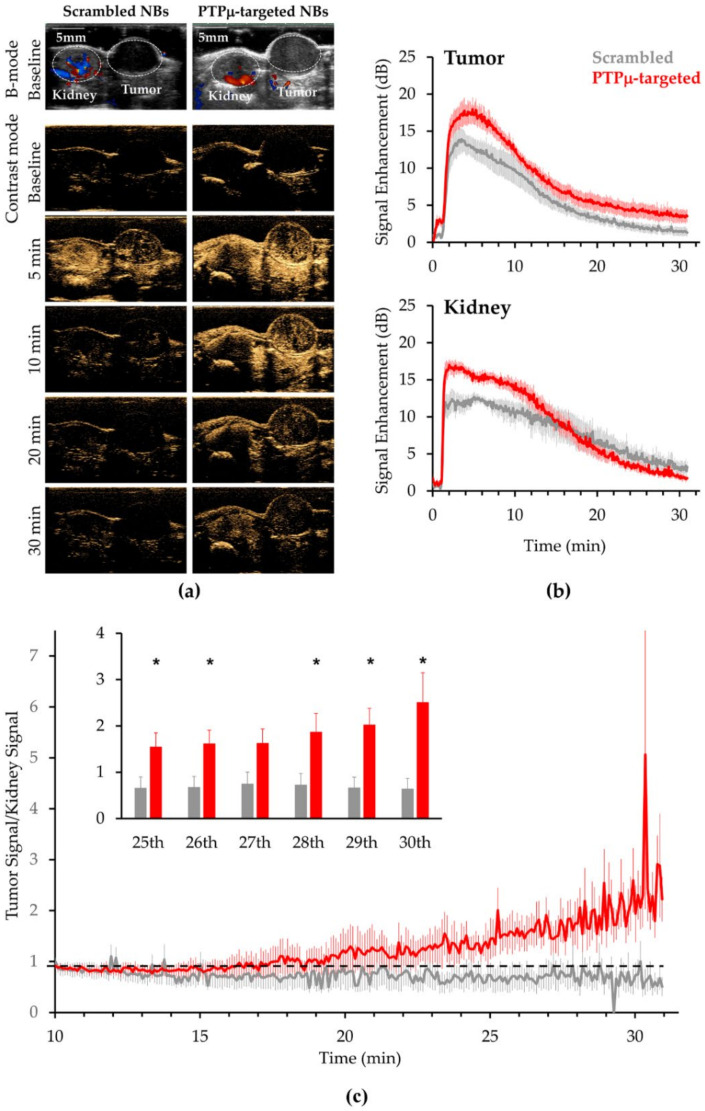
Ultrasound imaging of a representative tumor-bearing mouse treated with Scrambled and PTPµ-targeted NBs and time intensity curves from the tumor-bearing mouse cohort after administering Scrambled and PTPµ-targeted NBs. (**a**) In vivo US signal produced by the two NB types at various times. Baseline images in B-mode used for positioning and contrast mode images are shown for a representative mouse administered Scrambled NB (left) compared to PTPµ-targeted NB (right) along with contrast mode images at indicated times following the start of the scan. NBs were injected at a rate of 0.26 mL/min after acquiring a 1 min baseline. (**b**) The contrast harmonic signal measured over time in tumor (top plots) and kidney (bottom plots) in response to Scrambled-NB (gray) and PTPµ-targeted NBs (red). *N* = 6 for mice injected with Scrambled-NBs, *N* = 7 for mice injected with PTPµ-targeted NBs. Plots show mean signal intensity ± standard error. (**c**) Tumor signal normalized to kidney signal for the mice shown in panel (**b**) at later time points. PTPµ-targeted NBs showed relatively slower clearance from the tumor towards the end of the scan time compared to Scrambled NBs. Plots show mean normalized value ± standard error. Inset shows mean normalized values during the indicated one-minute intervals for the two NBs types (* *p* < 0.05 as indicated for 25th–26th and 28th–30th minutes, *p* = 0.05 for the 27th minute interval). During the 30th minute, the normalized PTPµ-targeted NB value was 3.9-fold higher than that of the untargeted NBs.

Mice bearing PTPµ biomarker-expressing flank tumors were prepared as described to use for comparing the in vivo characteristics of PTPµ-targeted and Scrambled NBs. After acquiring a standard B-mode image to properly align the imaging plane, the mode was switched to nonlinear contrast mode and, following a 1 min baseline acquisition, mice were injected with 200 µL of a given type of NB (2 × 10^11^ NB/mL) over 45 s and imaged for a total of 30 min. Figure 3a shows images for a representative mouse imaged with both NB types. The CEUS signal after NB injection occurred rapidly with both PTPµ-targeted and untargeted NBs and filling of both kidney and tumor 5 min after scan start (4 min after NB injection) was evident (Figure 3a). Mean time intensity curves (TICs) from a total of *N* = 6 mice treated with the Scrambled NB and *N* = 7 mice treated with the PTPµ-targeted NBs are shown in Figure 3b. Contrast enhancement in the tumor was apparent within 15 s of starting the NB injection and as shown in Figure 3b, peak tumor contrast occurred between 2 and 4 min of initiating the NB administration (3–5 min after the scan started) for both agents. Peak tumor signal occurred around 5 min for PTPµ-targeted NBs while peak tumor signal for Scrambled NBs occurred slightly earlier, at around 4 min. As shown in the upper panel of Figure 3b, PTPµ-targeted NB tumor signal cleared more slowly over the 30 min imaging time than that of the Scrambled NB (Figure 3b). The kinetics of signal enhancement in kidney for the targeted and non-targeted NBs were similar with peak signal occurring around 3 min and persisting out to 10 min (Figure 3b, lower panel). Significantly higher signal was detected in kidney of the tumor-bearing mice treated with the PTPµ-targeted NBs compared to the Scrambled NBs throughout this time (Figure 3b). These results suggest that the PTPµ biomarker may be present in kidneys. In contrast to tumor, the PTPµ-targeted NB signal declined more rapidly in kidney, starting around 12 min, compared to Scrambled NBs and both were near baseline levels by 30 min. To more closely examine the differences in PTPµ-targeted and untargeted NB clearance at later time points, normalized values were obtained by dividing the tumor signal by the kidney signal for each replicate and plotted (Figure 3c). As shown in Figure 3c, the tumor/kidney values for the PTPµ-targeted NBs were 0.9 ± 0.1 at 10 min and continued to increase to 2.5 ± 0.6 at 30 min. In contrast, the tumor/kidney values for the Scrambled NBs were 0.9 ± 0.1 at 10 min but decreased steadily to 0.6 ± 0.2 at 30 min. During the last 5 min of scan time, the tumor/kidney values were significantly higher in mice receiving the PTPµ-targeted NBs than in mice injected with the untargeted NBs, with the exception of the 27th min where *p* = 0.05, indicating relatively more tumor signal persisted in those mice (Figure 3c, inset panel).

**Figure 4:**
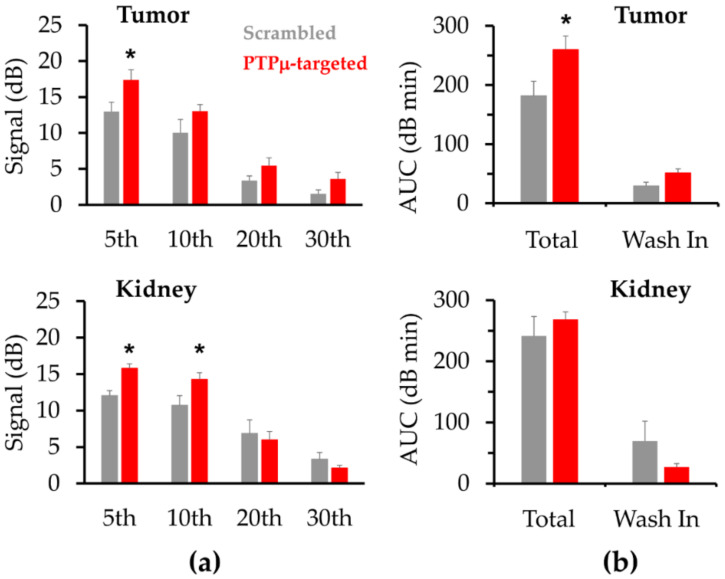
PTPµ-targeted NBs show greater contrast in tumors and are retained longer than Scrambled NBs. (**a**) The mean ± standard error (SE) signal intensity was calculated from the 12 echo power measurements collected for each 1-minute increment for the data represented as time intensity curves (TICs) in 3b. In tumor, the US signal produced by PTPµ-targeted NBs was significantly greater than that of the Scrambled NBs during the 5th through 7th minute increments. In kidney, the US signal produced by PTPµ-targeted NB was significantly greater than that of the Scrambled NBs from the 3rd through 10th minute increments. (**b**) Area under the curve (AUC) parameters obtained from the TICs shown in 3b. In tumor, PTPµ-targeted NBs had significantly higher total and AUC_wash-in_ compared to Scrambled NBs. In kidney, total AUC and AUC_wash-in_ were not significantly different between PTPµ-targeted NB and Scrambled NBs. Bars represent the mean ± SE of the AUC type indicated, (* *p* < 0.05).

CEUS signals are inherently noisy. To better assess relative signal intensities of the two NB types for each region of interest (ROI), the 12 echo power values collected over a given minute were averaged and compared for each one-minute interval. Using this approach, the peak PTPµ-targeted NB tumor signal of 17.4 ±1.4 dB, occurred during the 5th minute while the peak Scrambled NB tumor signal, 13.6 ± 1.6 dB, occurred during the 4th minute. The mean PTPµ-targeted NB tumor signal was more than 33% and significantly higher than the Scrambled NB signal from the 5th through 7th minute intervals. During these one-minute intervals, the mean PTPµ-targeted signals were 17.4 ± 1.4 dB, 17.1 ± 1.4 dB and 16.3 ± 1.3 dB compared to the corresponding Scrambled NB signals of 13.0 ± 1.3 dB (*p* < 0.05), 12.4 ± 1.4 dB (*p* = 0.03) and 11.9 ± 1.6 dB (*p* < 0.05). Figure 4a, upper panel, shows the mean tumor signals obtained from PTPµ-targeted and Scrambled NBs at one-minute intervals corresponding to 5, 10, 20 and 30 min. After peak enhancement, the tumor signal decreased for both types of NBs, but the PTPµ-targeted NBs persisted in the tumor for longer and cleared more slowly than the Scrambled NBs. During the 25th min, the mean US signal for the PTPµ-targeted NBs was 4.3 ± 1.0 dB and the US signal for the Scrambled NBs was 2.1 ± 0.6 dB. During the 30th minute, the mean US signal from the PTPµ-targeted NBs was more than 20% of the peak US intensity (3.6 ± 0.9 dB) while the US signal from the Scrambled NBs was about 11% of that at the peak (1.5 ± 0.5 dB). As shown in Figure 4a, lower panel, the PTPµ-targeted NB signal was also significantly higher than the Scrambled NB signal in kidney during the 5th and 10th minute intervals. The mean PTPµ-targeted signals were 15.8 ± 0.6 dB and 14.3 ± 0.3 dB, compared to the corresponding Scrambled NB signals of 12.0 ± 0.7 dB (*p* < 0.01) and 10.7 ± 1.3 dB (*p* < 0.05) for the 5th and 10th min, respectively. By 20 min, the PTPµ-targeted NB signal in kidney was less than that of the Scrambled NBs, although these values were not significantly different.

Parameters extracted from the aggregated TIC shown in Figure 3b provided additional insights into the nature of the PTPµ-targeted and Scrambled NBs enhancement and are depicted in Figure 4b and Table 1. For tumors, the total area under the curve (AUC) as well as the AUC_wash-in_ were significantly higher after PTPµ-targeted NB dosing, 260.1 ± 23.1 dB·min and 51.5 ± 6.8 dB·min, respectively, compared to tumors following Scrambled NB dosing, 181.8 ± 24.6 dB·min (*p* = 0.04) and 29.4 ± 6.4 dB·min (*p* = 0.04), respectively. Significantly higher AUC indicated more prolonged enhancement of the tumors by the PTPµ-targeted NB. AUC_wash-out_ was also higher in tumors treated with PTPµ-targeted NBs, but the values were not significantly different (Table 1). As shown in Table 1, other parameters evaluated between the two NB types included the peak intensity, peak time, time for the peak to decrease by 50% (*t*_50%_) and the wash-out rate. In contrast to comparisons of mean signals made over 1 min intervals described above, these TIC parameters were based on the data acquired every 5 s during the scan. Although the absolute maximum peak intensity values of the two NB types were not significantly different (Table 1), it is nonetheless important that the mean signals determined over three consecutive 1 min intervals were significantly higher in tumors following PTPµ-targeted NB treatment compared to tumors treated with Scrambled NBs. In kidney, the total AUC did not differ significantly between the PTPµ-targeted and Scrambled NBs, indicating that overall kidney enhancement by PTPµ-targeted NBs was not prolonged relative to the non-targeted NBs (Figure 4b, Table 1). Like total AUC, AUC_wash-in_ did not differ significantly in kidney between the two NB types, although AUC_wash-out_ was significantly higher following PTPµ-targeted NB treatment compared to Scrambled NBs (Table 1). As shown in Table 1, kidney enhancement by PTPµ-targeted and the non-targeted control NBs showed no significant differences in terms of peak time, *t*_50%_, or wash-out rate. In contrast to these parameters, the peak signal intensity in kidney achieved by PTPµ-targeted NBs was significantly higher than that obtained following Scrambled NB injection (Table 1).

**Figure 5:**
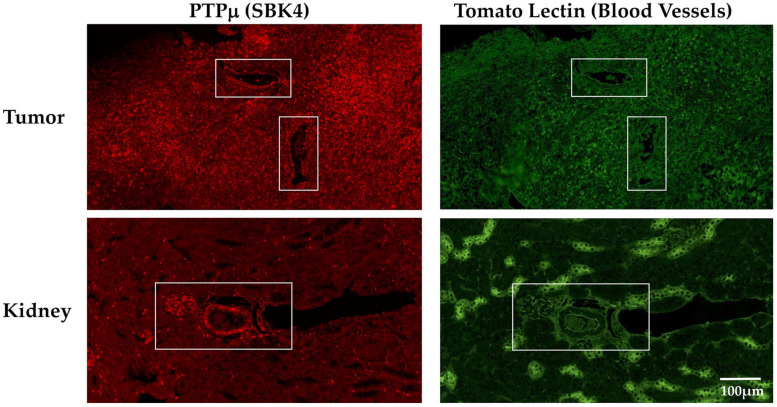
Localization of the PTPµ biomarker and endothelial cells in tumor and kidneys. Tumors and kidneys were obtained from mice bearing LN-229 flank tumors, fixed and stained with SBK4-TR, a peptide agent specific for the PTPµ biomarker and fluorescein isothiocyanate (FITC)-labeled tomato lectin. The top left panel shows high levels of the PTPµ-biomarker throughout the tumor, as well as on the surface of vascular structures (boxed areas). Diffuse tomato lectin staining (top right panel) was found throughout the tumor as well as on the same vascular surfaces (boxed areas) as the PTPµ biomarker. Less of the PTPµ biomarker was detected overall in the kidney (lower left panel), compared to tumor, but the vasculature showed high levels of the biomarker (boxed area). Tomato lectin stained vascular structures, similar to the bright SBK4-TR areas, as well as distinct cells throughout the kidney. All images were acquired at the same scale shown in the lower right panel.

The differences detected in CEUS mediated by the PTPµ-targeted and Scrambled NBs suggested vascular targeting. Therefore, we performed immunohistochemistry to determine the distribution of the PTPµ biomarker relative to endothelial cells in both tumor and kidney. LN-229 tumors and kidneys were harvested from tumor-bearing mice, formalin-fixed, processed and stained with reagents specific for the PTPµ biomarker and for N-acetylglucosamine, a carbohydrate group found in abundance on endothelial cells, using fluorescein isothiocyanate (FITC)-labeled tomato lectin (*Lycopersicon esculentum*) [31]. As shown in Figure 5, the PTPµ biomarker was present throughout the tumor and also appeared uniformly distributed on the surfaces of vascular structures (Figure 5, upper row, boxed areas). Tomato lectin labeled the same vascular structures (Figure 5, upper row, boxed areas) as SBK4-TR, the PTPµ biomarker specific agent, as well as other parts of the tumor. In kidney, the most intense SBK4-TR staining was of vascular structures similar to those recognized by the tomato lectin (Figure 5, bottom row, boxed area).

## 3. Discussion

For this study, we utilized NBs based on the highly stable formulation described by de Leon and colleagues [18], along with a PTPµ-targeting peptide to examine targeting and contrast enhancement in a tumor model relative to non-specific NBs. In vitro and in non-tumor bearing control mice, both NB types showed equivalent signal enhancement characteristics in heart, kidney and liver. In vivo, PTPµ biomarker-rich flank tumors showed more signal enhancement throughout the tumor and more sustained signal enhancement with the PTPµ-targeted NBs relative to the Scrambled-NB control agent. In these experiments the kidneys of tumor-bearing mice, but not non-tumor-bearing control mice, showed significantly higher nonlinear peak signal following treatment with PTPµ-targeted NBs compared to the Scrambled NBs. We have not previously observed any differences in kidney targeting or enhancement between PTPµ-targeted and Scrambled imaging agents in other imaging modalities such as MRI or fluorescence imaging (MJ, JAV, SBK, unpublished observations). It is possible that the combination of real time image acquisition and the nature of the NBs used in US allowed the detection of the PTPµ-biomarker in the kidneys of these tumor-bearing mice. The other methods mentioned (fluorescence imaging and MRI) require longer image acquisition times and use agents where the targeting peptide is in a 1:1 molar ratio with the moiety used for detection. In both of these modalities, the moiety conferring the signal (i.e., fluorophore or gadolinium chelate) provides a detectable signal both while binding to the tumor and after clearance into the kidney and eventually bladder. The total signal provided by the contrast agent will remain essentially constant but will be redistributed throughout the body as time elapses, eventually accumulating in the renal system until the mouse wakes up and can void. Under these circumstances, it is possible that specific interactions of both fluorophore-peptides and gadolinium-chelate-peptides with kidney are obscured by the clearance of either excess agent or agent that has bound and released from tumor. In contrast, US contrast agents such as the PTPµ-targeted NBs used in these studies only provide a detectable signal while they are intact NBs. The signal provided by the administered contrast agent, in this case PTPµ-targeted NBs, continually decreases as NBs are destroyed in response to the pressures encountered in the ultrasound field. Remnants of NBs clearing through the renal system after binding to the tumor will not provide a detectable signal with the potential to obscure a kidney-specific signal. Thus, these CEUS studies have provided an opportunity to detect a potential kidney interaction with PTPµ-targeted agents that we were unable to detect in our previous studies. In addition, each individual NB has many targeting peptides, unlike the fluorophore-peptide or gadolinium-chelate-peptides used in our previous studies. The binding characteristics of NBs may differ from those of the other agents and facilitate the visualization of kidney binding. To better understand what may account for the greater kidney enhancement with PTPµ-targeted NBs compared with Scrambled NBs, we looked for the presence of the PTPµ biomarker in kidneys of tumor bearing mice. As shown in Figure 5, the PTPµ biomarker and endothelial cells were found in close proximity in particular areas of the kidney. It is possible that the circulation of PTPµ-targeted NBs through the kidney facilitates the interaction of PTPµ-targeted NBs with the PTPµ biomarker in this organ, leading to enhanced US signals shortly after injection of the PTPµ-targeted NBs. Soluble forms of some cell adhesion molecules can circulate at detectable levels in many types of cancer patients [32,33,34,35,36]. Since the PTPµ biomarker is formed in response to proteolytic events, it is possible that it too might be taken up by the circulatory system in a soluble form. As a clearance organ, the kidney could potentially filter and concentrate any soluble PTPµ biomarker released from PTPµ-enriched tumors resulting in the kidney being recognized by PTPµ-targeted NBs. We note that despite the significantly higher nonlinear signal observed in the kidneys of these tumor-bearing mice following treatment with the PTPµ-targeted NBs, there was no significant difference in AUC derived from TICs of the two NB types suggesting that the PTPµ-targeted NBs were not retained to a significantly greater extent in the kidney relative to the control agent. Nonetheless, this is an intriguing finding that we plan to study further.

These results are consistent with prior studies using a similar NB formulation but targeted to the prostate specific membrane antigen (PSMA) [11]. Specifically, in both studies, the NBs targeted to PSMA show similar slower washout kinetics and greater tumor retention at the end point of the study compared to NBs without a targeting ligand. However, in contrast to the PSMA biomarker, which is expressed only on prostate cancer cells, PTPµ is expressed on endothelial cells and specifically found at cellular junctions which mediate paracellular permeability and potentially extravasation [37,38,39,40]. We hypothesize that this difference in biomarker expression is responsible for the distinctly higher peak intensity of the PTPµ-targeted NBs compared to untargeted NBs. When imaging PSMA, this difference is not seen and peak intensity of both types of bubbles is nearly identical. This could be an exciting indicator that targeted NBs can, in real time, recognize biomarker distribution and display binding kinetics. Furthermore, the binding of NBs to the endothelium would likely reduce the fraction of NBs which extravasate into the tumor parenchyma. This could be the reason for a relatively smaller difference in the targeted versus untargeted NB retention in this model compared to PSMA-targeted NBs. This is also supported by the latent increase in tumor signal compared to kidney signal observed for the PTPµ-targeted NBs. In this model, the tumor signal does not increase perceivably until 15 min after bubble injection. In contrast, the same parameter in PSMA-targeted NBs shows a rapid increase after 5 min. This may be a result of decreased endothelial bubble binding in the latter portion of the scan. Taken together, these data demonstrate that PTPµ-targeted NBs can effectively target PTPµ and persist in tumors. In addition to its use in echocardiography and other settings, US is often the first imaging modality employed in the diagnosis of ovarian cancer [41]. We have recently detected high levels of the PTPµ biomarker in several types of gynecological cancers including high grade serous ovarian cancer [42]. Our future plans include utilizing mouse models of multiple types of cancer along with our PTPµ-targeted NBs to expand these findings and to utilize PTPµ-targeted NB as theranostics.

In summary, PTPµ-targeted NBs demonstrated excellent stability both in vitro and in vivo. In models of tumor imaging in vivo, PTPµ-targeted NBs showed increased contrast throughout the tumor and greater peak contrast when compared to non-targeted Scrambled-NBs. Enhanced signal was detectable in tumor within 15 s of initiating the injection of either NB type, but PTPµ-targeted NBs showed significantly higher signal compared to Scrambled NBs at peak contrast which occurred throughout a 5–7 min interval following the start of the scan. At later time points the contrast enhanced signal was also higher using PTPµ-targeted NBs relative to the control agent. In addition, the total AUC value derived from the PTPµ-targeted TIC was significantly higher than the total AUC from Scrambled TIC, further demonstrating effective tumor enhancement by the PTPµ-targeted NBs. Based on these studies, the use of PTPµ-targeted NBs for diagnostic and theranostic use warrants further investigation.

## 4. Materials and Methods

### 4.1. Materials

Nanobubbles were made from a combination of 1,2-dibehenoyl-sn-glycero-3-phosphocholine (DBPC), 1,2-dipalmitoyl-sn-glycero-3-phosphate (DPPA) and 1,2-dipalmitoyl-sn-glycero-3-phosphoethanolamine (DPPE), all purchased from Avanti Polar Lipids, Pelham, AL, USA, along with 1,2-distearoyl-sn-glycero-3-phosphoethanolamine-N-(methoxy(polyethylene glycol)-2000) (ammonium salt) (DSPE-mPEG 2000) from Laysan Lipids, Arab, AL, USA. The lipids were dissolved in propylene glycol (PG, Sigma Aldrich, Milwaukee, WI, USA), glycerol (Acros Organics) and Gibco phosphate buffered saline (PBS, pH 7.4, 1×) from Thermo Fisher, Waltham, MA, USA.

### 4.2. Peptide-Lipid Conjugation

The SBK2 (GEGDDFNWEQVNTLTKPTSD) and Scrambled (GTQDETGNFDWPVSEDLNKT) peptides were synthesized on a CS Bio CS336X Synthesizer as described previously [43] using Fmoc-protected amino acids purchased from either Chem-Impex, Wood Dale, IL, USA or Aapptec, Louisville, KY, USA. An N-terminal cysteine was included along with a six amino acid spacer (GGSGGS) to allow conjugation of the peptides to 1,2-distearoyl-sn-glycero-3-phosphoethanolamine-N-(methoxy (polyethylene glycol)-2000)-maleimide (DSPE-PEG-MAL) Laysan Bio, Arab, AL via a thiol-maleimide reaction. Peptides were conjugated to DSPE-PEG-MAL by thoroughly mixing peptide with lipid in PBS, pH 8.0 in a 1:2 molar ratio and incubating the reaction for 2 h at room temperature. The reaction mixture was checked by reverse phase high performance liquid chromatography (HPLC) on a Shimadzu HPLC system equipped with an SPD-20A Prominence UV/visible detector and monitored at 220 nm and 254 nm using a Luna 5 µ C18(2) 100A column (250 mm × 46 mm × 5 mm, Phenomenex, Torrance, CA) at a flow rate of 0.8 mL/min (Figure S1). The mobile phase was 10–90% acetonitrile versus water with 0.1% trifluoroacetic acid over 30 min [11]. After confirming that no unreacted peptide remained in the mixture, the reaction was lyophilized. The lyophilized product was dissolved in PBS to make a stock solution of 1 mg/mL concentration to use in making the nanobubbles. Matrix-assisted laser desorption/ionization time-of-flight mass spectroscopy (MALDI-TOF-MS) was also performed to confirm the successful conjugation of the peptides to DSPE-PEG-MAL using 20 mg/mL 2,5-dihydroxybenzoic acid in 50% acetonitrile/50% water with 0.1% trifluoroacetic acid as matrix. These analyses were performed by the Center of Materials and Sensor Characterization at the University of Toledo, Toledo, OH, USA on a Bruker UltrafleXtreme MALDI TOF/TOF Mass spectrometer (Figure S2).

### 4.3. Nanobubble Formulation 

Nanobubbles were formulated as previously described [11,44]. A lipid mixture consisting of 6.1 mg of DBPC, 2 mg of DPPE, 1 mg of DPPA and 1 mg of mPEG-DSPE was dissolved in 0.1 mL of propylene glycol in a sonicating water bath heated to 80 °C. For PTPµ-targeted or Scrambled NBs, 75 µg of either DSPE-PEG-SBK2 or DSPE-PEG-Scrambled from the stock solutions described above were added to the DBPC/DPPE/DPPA/mPEG-DSPE cocktail. In a separate container, 0.8 mL of PBS (pH 7.4) and 0.1 mL of glycerol were combined and warmed to 80 °C and then added to the lipid solution. After 10 min in a room temperature sonicating water bath, the solution was placed in a 3 mL headspace vial, capped with a rubber stopper and sealed with an aluminum cap. The air within the vial headspace was replaced with octafluoropropane gas (AirGas, Cleveland, OH, USA). Bubbles were formed by agitation of the vial with a VialMix mechanical shaker for 45 s. After centrifugation for 5 min at 50× *g*, nanobubbles were collected with a syringe (21 G × 1 in.) from the bubble fraction within 0.5 cm of the stopper, as described previously [44].

### 4.4. In Vitro Nanobubble Characterization

The nanobubble size and concentration were measured using resonant mass measurement (RMM) (Archimedes, Malvern Panalytical Inc., Westborough, MA, USA) [44]. A nanosensor, which can measure particle size between 100 nm and 2 µm, was used to measure the particles and calibrated using National Institute of Standards and Technology (NIST) traceable 565 nm polystyrene bead standards (ThermoFisher 4010S, Waltham, MA, USA). The NBs were diluted (1:1000) with PBS, pH 7.4 prior to measurement.

In vitro US stability of the nanobubbles was assessed using an AplioXG SSA-790A ultrasound instrument (Toshiba Medical Imaging Systems, Ottaware-Shi, Japan) and a custom-made 1.5% (*w*/*v*) agarose mold with a triple channel (L × W × H per channel = 5 mm × 3 mm × 6 mm) and imaged for 8 min. Acquisition parameters included contrast harmonic imaging (CHI) with 12.0 MHz harmonic frequency, 0.1 mechanical index (MI), 65 dB dynamic range and 70 dB gain. The ultrasound signal was determined using the pre-loaded quantification software (CHI-Q). Samples were diluted 1:100 in PBS, pH 7.4 prior to imaging.

### 4.5. Mice and Experimental Tumor Models

National Institutes of Health (NIH) athymic nude female mice (NCr-nu/+, NCr-nu/nu) were bred in the Case Western Reserve University Athymic Animal Core Facility. Tumors were initiated in mice at approximately 6 weeks of age. Human LN-229 tumor cells were chosen for these studies as a model tumor type expressing the PTPµ biomarker and have been used in previous imaging studies to examine PTPµ-targeted agents [25,30]. These cells were obtained from the American Type Culture Collection (Manassas, VA, USA) and cultured as previously described [25]. Cells were mixed with Matrigel Matrix (Corning, Corning Inc., Corning, NY, USA) and 2 × 10^6^ cells were injected into the right flank of each mouse. Mice were used for in vivo experiments when tumors were between 44 and 58 d old. The mean (SE) tumor age for mice receiving targeted NBs was 50.3 (2.2) d and for mice receiving the untargeted NBs, the mean (SE) was 50.3 (4.5) days. Tumor volumes calculated using the formula (length × width^2^)/2 were 457.8 (116.8) mm^3^ and 512.5 (95.7) mm^3^ for the tumors used with PTPµ-targeted and Scrambled NBs, respectively, and were not significantly different using a two-tailed *t*-test (*p* = 0.78). For some experiments, athymic nude mice without tumors were used for in vivo experiments between the ages of 6 and 8 weeks. 

### 4.6. In Vivo US Imaging

An Aplio XG SSA-790A Toshiba Medical Imaging Systems (now Hitachi) clinical ultrasound imaging system equipped with a 12 MHz linear array transducer was used for these experiments. For US imaging of tumor-bearing mice, animals were anesthetized with 2–3% isoflurane with 1 L/min oxygen and positioned on their left lateral sides on a 37 °C platform. A PLT-1204BT ultrasound probe was positioned along the longitudinal axis to capture signals from both the tumor and kidney and clamped into position using B-mode imaging information. Changes in tissue contrast over time were measured using contrast harmonic imaging (CHI) with harmonic receive frequency set to 12.0 MHz, mechanical index (MI) set to 0.10, a dynamic range of 65 dB, gain at 70 dB and the imaging frame rate set to 0.2 frame/s. The images were acquired in the raw data format [45]. Baseline values were acquired for 60 s before intravenous administration of 200 µL (2 × 10^11^ NB/mL) of either the PTPµ-targeted NBs or the Scrambled NBs via a tail vein catheter. NB injections occurred at the rate of approximately 0.26 mL/min. Animals were imaged for 30 min following injection. Most animals received both types of NBs in separate scans in random order and these in vivo experiments were separated by 5–10 d. In total, *N* = 6 for mice treated with the Scrambled NBs and *N* = 7 mice were scanned with the PTPµ-targeted NBs.

In vivo experiments in non-tumor bearing mice (*N* = 3) were performed in a similar manner with the exception that animals were positioned on their backs to allow the probe to capture signal from heart, liver and kidney. For these experiments, 100 µL of both types of NB were administered on a given day but separated by at least 60 min. After injection of one NB type, the animal was imaged for 30 min, subjected to 3 high intensity ultrasound bursts to destroy NBs remaining in circulation and maintained in position for another 30 min to allow any residual NBs to clear before administering the other NB type and monitoring for 30 min.

Software provided by Toshiba was used to process the acquired linear raw data images as has been described previously [11,45]. ROIs outlining the kidney and tumor were drawn on each mouse on the contrast mode image using the B-mode image as reference to avoid regions of skin and major blood vessels and the mean echo power value as a function of time was determined for each ROI type and exported to Microsoft Excel. Twelve echo power values were obtained per minute, corresponding to one value every 5 s (0.083 min). The lowest signal value obtained for each ROI during an imaging session was used as the baseline value for that ROI and subtracted from the other echo power values recorded during the scan to generate the time-intensity curves (TIC). To examine NB clearance at later time points, tumor signal was normalized to the signal in kidney for each mouse by dividing the tumor signal by the kidney signal at each time point. The means (SEs) were plotted for each cohort. For comparison of US signals at different time intervals, the mean of the 12 echo power signals acquired over a given minute for each animal were used to represent the US signal for that animal during that minute. As indicated below, significance was determined using two-tailed *t*-tests.

### 4.7. Histology

Tumors and kidneys were harvested from mice with approximately 40-d old LN-229 flank tumors, fixed in 10% neutral-buffered formalin (Sigma-Aldrich, St. Louis, MO, USA) and paraffin embedded before making 5 µm tissue sections. Tissue sections were stained for the PTPµ biomarker using the SBK4 peptide conjugated to Texas Red as has been previously described [28], in parallel to sections stained with the control Texas Red-conjugated Scrambled peptide. FITC-labeled tomato lectin (Vector Laboratories, Inc., Burlingame, CA, USA), which binds N-acetylglucosamine present on endothelial cells [31], was used according to the manufacturer’s directions to stain blood vessels. After staining, sections were mounted with Vectashield Hard Set Mounting Medium (Vector Laboratories, Inc. Burlingame, CA, USA), coverslipped and imaged with a Hamamatsu Nanozoomer S60 Slide Scanner (Hamamatsu Photonics, K.K., Bridgewater, NJ, USA).

### 4.8. Statistical Analysis

Microsoft Excel and OriginLab Origin were used for graphing and statistical analyses. Where indicated, unpaired two-tailed Student’s *t*-tests were used to compare the two groups. Various TIC parameters such as area under the curve (AUC), time to peak, peak intensity and wash-out rate were calculated in Origin and have been used previously to describe and assess contrast enhanced US [2,11,46]. Briefly, peak intensity is the maximum signal measured in an ROI during a scan, time to peak is the amount of time in minutes from the initiation of the scan to the peak signal, AUC_wash-in_ represents the AUC from injection start to the peak signal and AUC_wash-out_ is the AUC from peak signal to scan end. Wash-out rate was determined by measuring the slope of the TIC between maximum peak intensity to half peak height. Peak intensity and time to peak values extracted from the TIC curves and based on the data acquired every 5 s vary slightly from similar parameters determined by averaging the signal over one-minute increments as described above used to compare data at minute intervals. Data are presented as mean ± standard error (SE). The number of replicates is indicated for each experiment.

## Figures and Tables

**Table 1:** Summary of parameters extracted from time intensity curves for tumor and kidney.

	Scrambled Mean (± SE)	PTPµ-Targeted Mean (± SE)	*p*-Value
Tumor			
AUC (dB·min)	181.8 (24.6)	260.1 (23.1)	0.04 *
AUC_wash-in_ (dB·min)	29.4 (6.4)	51.6 (6.8)	0.04 *
AUC_wash-out_ (dB·min)	152.4 (22.8)	208.5 (22.6)	0.11
Peak intensity (dB)	15.2 (1.5)	18.8 (1.6)	0.13
Peak time (*t*_max_) (min)	3.9 (0.7)	5.2 (0.7)	0.23
Time to reach 50% of peak (*t*_50%_) (min)	12.3 (1.5)	15.1 (2.7)	0.40
Wash-out rate (ΔdB/min)	−1.0 (0.3)	−1.1 (0.2)	0.84
Kidney			
AUC (dB·min)	241.6 (31.6)	268.9 (11.8)	0.41
AUC_wash-in_ (dB·min)	69.6 (32.3)	26.8 (5.9)	0.19
AUC_wash-out_ (dB·min)	172.0 (28.3)	242.1 (11.9)	0.03 *
Peak intensity (dB)	15.5 (1.1)	18.2 (0.6)	0.04 *
Peak time (min)	7.4 (2.9)	2.9 (0.5)	0.13
Time to reach 50% of peak (*t*_50%_) (min)	17.8 (3.2)	16.5 (1.4)	0.69
Wash-out rate (ΔdB/min)	−0.6 (0.1)	−0.5 (0.1)	0.44

* Significantly different (*p* < 0.05).

## Data Availability

The data that support the findings of this study are available from the corresponding author upon reasonable request.

## Supplementary Materials

The following are available online at https://www.mdpi.com/1422-0067/22/4/1983/s1. Figure S1: Conjugation of the Scrambled and SBK2 peptides to the lipid DSPE-PEG-maleimide, Figure S2: MALDI-TOF MS analysis of DSPE-PEG-SBK2 and DSPE-PEG-Scrambled conjugates.
