# Supplementary material for: Ultrasound-Based Molecular Imaging of Tumors with PTPmu Biomarker-Targeted Nanobubble Contrast Agents
Source: Int J Mol Sci. 2021 Feb 17;22(4):1983. doi: 10.3390/ijms22041983 (PMC7922223; doi:10.3390/ijms22041983)
Supplement: Supplementary file 1 [file ijms-22-01983-s001.pdf]

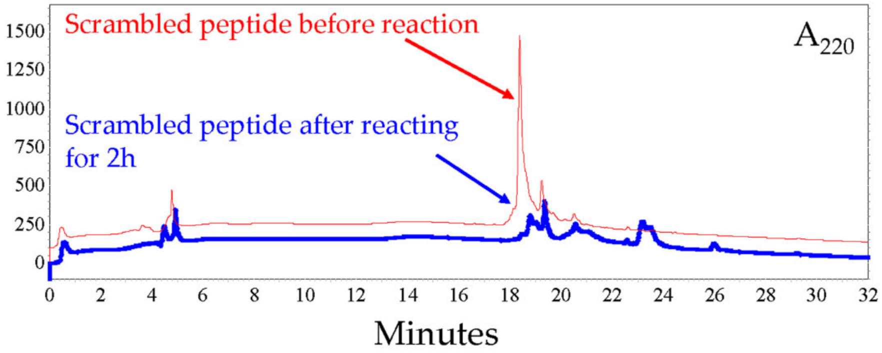

(a)

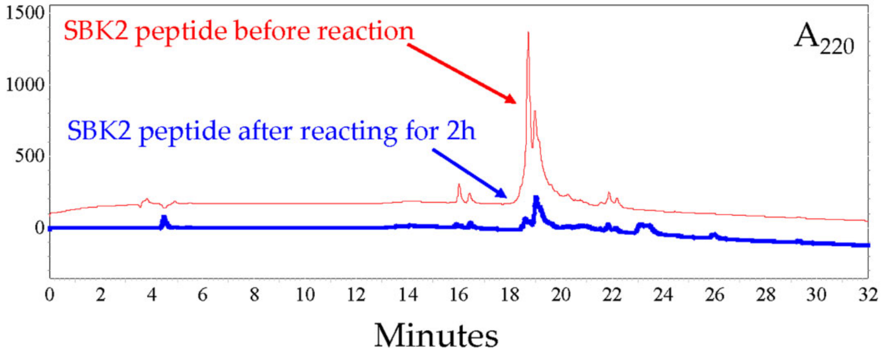

(b)

Figure S1. Conjugation of the Scrambled and SBK2 peptides to the lipid DSPE-PEG-maleimide. Chromatograms are shown for representative reactions demonstrating the successful conjugation of the Scrambled peptide (panel a) and the SBK2 (PTPμ targeting) peptide (panel b) to DSPE-PEG-maleimide. For each reaction, 5 μL injections of a 1:4 dilution of the reaction mixture at the start (red tracing) and after 2 h (blue tracing) are shown. For both reactions, only trace amounts of either peptide (RT = ~18 min) remained after 2 h indicating that both the Scrambled and SBK2 peptides had been successfully conjugated to the lipid.

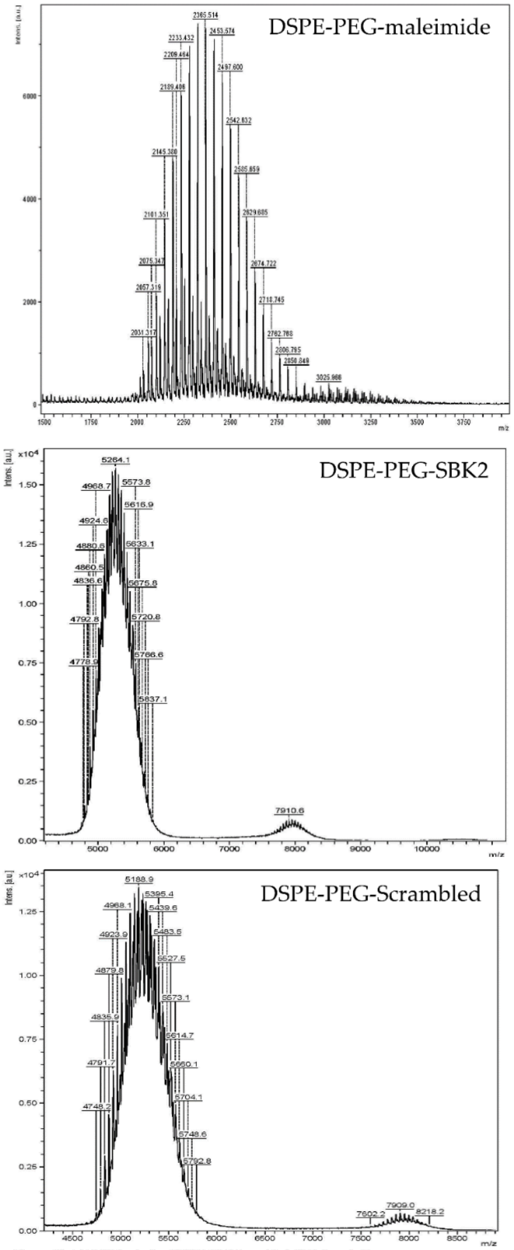

Figure S2. MALDI-TOF MS analysis of DSPE-PEG-SBK2 and DSPE-PEG-Scrambled conjugates. MALDI-TOF MS analysis of the polydisperse DSPE-PEG-maleimide shows a cluster of peaks around 2300 Da (top panel). The molecular weight (MW) of both the SBK2 and Scrambled peptides is 2758.82 Da. As shown in the middle panel for the DSPE-PEG-SBK2 conjugate, and in the lower panel for the DSPE-PEG-Scrambled conjugate, the conjugates each show a cluster of peaks around 5200 Da. The increased MW of the polydisperse species shown in the middle and lower panels confirms the successful conjugation of both the SBK2 and Scrambled peptides to DSPE-PEG-maleimide.
